# GIS-based spatio-temporal analysis of rainfall trends under climate change in different agro-ecological zones of Wolaita zone, south Ethiopia

**DOI:** 10.1016/j.heliyon.2024.e33235

**Published:** 2024-06-19

**Authors:** Elias Bojago, Ayele Tessema, Innocent Ngare

**Affiliations:** a Department of Environmental Science, College of Natural and Computational Sciences, Wolaita Sodo University, P.O. Box 138, Wolaita Sodo, Ethiopia; b Faculty of Environment, Gender and Development Studies, Hawassa University, P.O.Box 05, Hawassa, Ethiopia; c School of Agriculture and Environmental Sciences, Kenyatta University, P.O Box 00100, 43844, GPO, Nairobi, Kenya

**Keywords:** Agro-ecology, Kriging-interpolation, Mann-kendall, Rainfall, Sen's slope, Spatiotemporal analysis, Wolaita zone

## Abstract

Understanding the spatiotemporal dynamics of climatic conditions within a region is paramount for informed rural planning and decision-making processes, particularly in light of the prevailing challenges posed by climate change and variability. This study undertook an assessment of the spatial and temporal patterns of rainfall trends across various agro-ecological zones (AEZs) within Wolaita, utilizing data collected from ten strategically positioned rain gauge stations. The detection of trends and their magnitudes was facilitated through the application of the Mann–Kendall (MKs) test in conjunction with Sen's slope estimator. Spatial variability and temporal trends of rainfall were further analyzed utilizing *ArcGIS10.8 environment and XLSTAT with R programming tools*. The outcomes derived from ordinary kriging analyses unveiled notable disparities in the coefficient of variability (CV) for mean annual rainfall across distinct AEZs. Specifically, observations indicated that lowland regions exhibit relatively warmer climates and lower precipitation levels compared to their highland counterparts. Within the lowland AEZs, the majority of stations showcased statistically non-significant positive trends (p > 0.05) in annual rainfall, whereas approximately two-thirds of midland AEZ stations depicted statistically non-significant negative trends. Conversely, over half of the stations situated within highland AEZs displayed statistically non-significant positive trends in annual rainfall. During the rainy season, highland AEZs experienced higher precipitation levels, while the south-central midland areas received a moderate amount of rainfall. In contrast, the northeast and southeast lowland AEZs consistently received diminished rainfall across all seasons compared to other regions. This study underscores the necessity for the climate resilient development and implementation of spatiotemporally informed interventions through implementing region-specific adaptation strategies, such as water conservation measures and crop diversification, to mitigate the potential impact of changing rainfall patterns on agricultural productivity in Wolaita.

## 1 Introduction

Global climate change and unpredictability have exacerbated irregular rainfall in many places of the world [1,2]. Rainfall plays an integral role in shaping the planet's climate and water cycle through hydrological, ecological, and biochemical mechanisms [3,4]. Rising global temperatures directly impact where and when rain and snow fall each year [5,6]. Climate patterns vary widely across different zones owing to dissimilar underlying climatic conditions, diverse causal factors, and area-specific quirks [7]. Therefore, accurately tracking rainfall amounts is essential for properly managing freshwater supplies, hydrology projects, farming practices, and land usage, including flood and drought risk assessments [8]. Gauging trends and fluctuations in rainfall by region are thus of utmost importance. Monitoring how much moisture makes it to Earth in a given place at a certain time helps communities prepare for water-related hazards and harness water resources, core to human and environmental well-being [9].

Ethiopia remains highly vulnerable to climate change due to its mountainous landscape, erratic weather patterns, and insufficient adaptation strategies [2]. According to experts, a few countries have more episodes of severe weather events. Different scholars were observed how climate change has altered the ebb and flow (periodic fluctuations) of rainfall across diverse regions of Ethiopia [[10], [11], [12]]. This fluctuation endangers water provision and triggers an assortment of hydrological and meteorological disasters. Meanwhile, the country's diversified topography and demographic diversity further complicate coping and relief efforts for a population reliant on existing natural assets [9,13]. Going forward, bolstering resilience at the community level through collaborative projects and capacity-building will prove pivotal in enduring contemporary environmental unpredictability and safeguarding livelihoods [14].

Ethiopian agriculture remains a critical part of the national economy; with it determines percent's out 40 % of gross domestic product (GDP) (https://www.usaid.gov/ethiopia/agriculture-and-food-security). Eighty percent of Ethiopia's exports are derived from agricultural production, and around 75 % of the country's labor force is unemployed as result [15]. In agriculture more than 70 % of the total labor force finds employment and the industry's performance is positively correlated with rainfall. Therefore, rainfall shortages can cause famine [16,17]. Ethiopia experiences three distinct seasons: (i) June–September, the main rainy season known locally as “*Kiremt*”; (ii) February–May, the short rainy season called “*Belg*”; and (iii) October–January, the dry season referred to as “*Bega*.” Satellite imaging provides valuable insights into the rainfall patterns of the Kiremt (main rainy season) and Belg (short rainy season) [[18], [19], [20]]. Water-resource potential will also benefit from an improved understanding of these weather patterns in the countryside and season rainfall is important to improve agricultural productivity in the country [[19], [20], [21]], but also the planning and management waterworks systems use and interface with variable water flows every month in relation to long term water demand as well [22]. Kiremt, locally known as Meher [23,24], is the primary cropping season rain and is more consistent across most parts of the country compared to the rainfall during the Belg and Bega seasons (the post-harvest rainy periods in October and January) [23,25]. An absence of rainfall and the resulting variations affect the entire country's output from agriculture. A better understanding of these implications is capable of being enhanced even more by combining hydrodynamic and climate measurements [26,27].

High temporal and spatial variability in Ethiopia's Belg season rainfall has an impact on household level food security and Belg season crops [28,29]. The changes and alterations observed in Belg season rainfall patterns are anticipated to lead to severe drought conditions, significantly impacting socioeconomic welfare and environmental resources [30]. Given the extensive research on rainfall and temperature variability in Ethiopia across different temporal and spatial scales, our study seeks to summarize our findings in relation to those of previous investigations. Numerous studies have explored Ethiopian rainfall and temperature trends. Noteworthy among these are the works of Wagesho et al. [31], Gummadi et al. [32], and Alemayehu and Bewket [33]. However, despite the wealth of research, a clear and consistent pattern regarding Ethiopian rainfall remains elusive in Wolaita zonal level.

Their overall finding was that studies on rainfall variability and trends in Ethiopia did not reveal a clear pattern. The reasons for this could be as follows: (i) topography influences trends in annual and seasonal rainfalls [[34], [35], [36]]; (ii) trend analysis is highly sensitive to data quality, the selection of study periods and stations taken into consideration by the various studies [[37], [38], [39]]; (iii) the Inter Tropical Convergence Zone (ITCZ) oscillates north-south, the El Nino-Southern Oscillation (ENSO) phenomenon [[40], [41], [42]] and variations in sea surface temperatures (SSTs) a face mask trends [[43], [44], [45]]; and (iv) Large-scale investigation of temperature and rainfall variability and trends has been done previously [46], and has resulted in a variety of change patterns. The seasonal and annual rainfall totals in Ethiopia have been shown to be declining, despite the observed discrepancies [10,35,37,[47], [48], [49], [50]]. Environmental modelling, flood risk management, agricultural planning, and climate change adaptation all depend on climate analysis conducted at appropriate timescales [[51], [52], [53]].

Many researchers i.e., Worku et al. [54]; Bewket & Conway, [55]; Degefu & Bewket, [56]; Sahilu et al. [57], advocated for more AEZ-based local-level studies on rainfall variability and trends, as large-scale studies often mask spatial and temporal differences. Due to the country's diverse microclimates, ongoing investigation into rainfall variability is essential across AEZs. This study examines how AEZs affect the spatial variability and temporal trends of rainfall distributions within the Wolaita Zone.

This study uses 34 years of station monthly rainfall data covering the whole Wolaita zone (1987–2021), which spatially covers the zone, to examine the spatiotemporal patterns of rainfall in the southern region of Ethiopia. In countries like Ethiopia, where the economy heavily depends on rain-fed smallholder agriculture, timely and accurate climate information is crucial for sustainable climate risk management and agricultural practices [[58], [59], [60]]. Improved understanding of local long-term annual and seasonal rainfall variations is advantageous for adaptation measures [61].

This study aims to explore rainfall trends under climate change in the Wolaita zone through GIS-based spatio-temporal analysis. The hypothesis is that significant differences exist in rainfall patterns across various AEZs in Wolaita, and these patterns are evolving over time due to climate change. By studying spatiotemporal historical rainfall tendencies, this study attempts to provide significant insights into the influence of climate change, as well as flood and drought management to check crop failures across different AEZs of Wolaita.

The findings from local-level investigations, such as those reported in this article, are crucial for developing effective climate change adaptation strategies in agriculture. This approach contrasts with the typical coarse-scale national-level analysis, which often guides local decisions. Previously, no studies have examined the spatial variability and temporal patterns of rainfall across the three AEZs in the study area. This research addresses gaps in understanding seasonal patterns, both spatial and temporal trends of rainfall across AEZs of Wolaita.

Grasping the dynamics of rainfall trends and their spatial variability is essential for identifying vulnerable areas, planning adaptation measures, and enhancing the resilience of agricultural systems. Such understanding necessitates a context-specific approach, which this study emphasizes by focusing on the diverse AEZs within the Wolaita zone. This study's findings may guide future investigation and the creation of national and regional policies, contributing to the growing body of knowledge about the consequences of environmental change on Ethiopian agriculture. This study's practical application is to inform policymakers, researchers, and farmers about the hazards and opportunities connected with changing rainfall patterns in the Wolaita zone. Understanding the spatial and temporal based analysis of rainfall allows stakeholders to build targeted interventions and adaptation measures to improve agricultural systems' resilience to climate change.

## 2 Methodology

### 2.1 Description of the study area

**Fig. 1:**
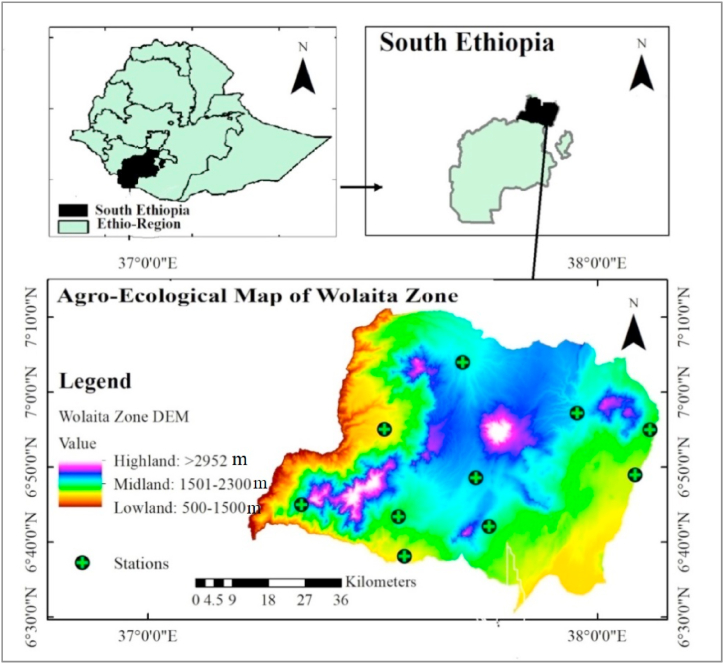
Map of the study area.

In south Ethiopia, Wolaita zone is located between latitudes 6°4′ N and 7°1′ N and longitudes 37°4′ E and 38°2′ E. The zonal seat, Wolaita Sodo, is situated 170 km away from Addis Ababa, the capital city of Ethiopia. It is composed of 16 woredas and 10 administrative towns. The zone covers 35 %, 56 %, and 9 % of the region, respectively, and is separated into three altitudinal zones: Kolla (500–1500 masl), Woina-dega (1500–2300 masl), and Dega (>2300 masl). The zone's altitude extends from 700 to 2900 m above sea level [62] (Fig. 1).

### 2.2 Sampling procedure

**Fig. 2:**
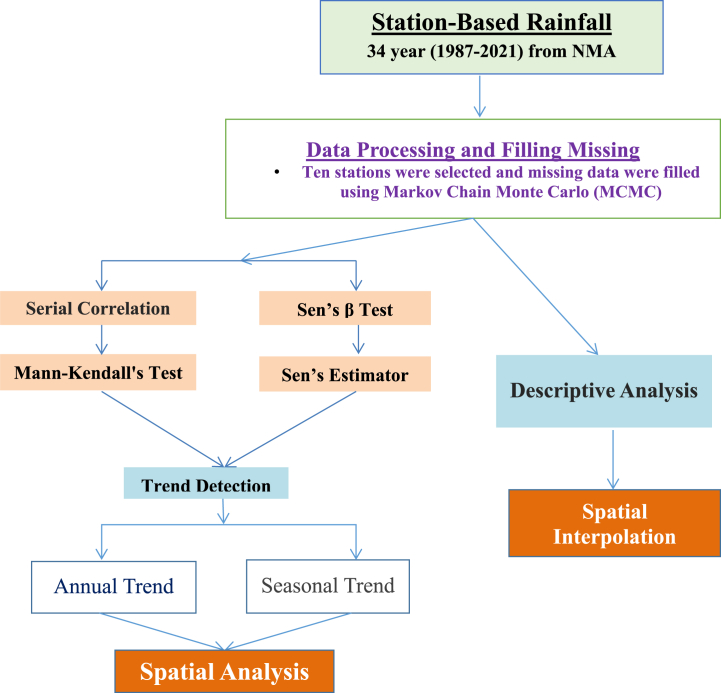
Methodological framework of the study.

The data from each station were averaged to generate representative data points for the entire study area. Seasonal trend analysis considered three seasons according to the NMA classification: Kiremt (summer season, from June to September), Belg (autumn-like season, from February to May), and Bega (dry season, from October to January) [92]. Fig. 2 illustrates the methodology used in this study.

The study's necessary groups were selected using a stratified random sampling approach, treating the AEZs as strata. A random sample was drawn from each stratum according to the proportion of each AEZ. Specifically, the lowland, midland, and highland AEZs constitute approximately 35 %, 56 %, and 9 % of the total area, respectively. Based on this distribution, four stations from the midland AEZ, three from the lowland AEZ, and two from the highland AEZ were randomly assigned (Table 1).

**Table 1:** List of weather stations used for long term rainfall.

Names of Stations	Latitude	Longitude	Altitude	Periods	Missing (%)
Areka	7°04′00.0″N	37°42′00.0″E	1774	1987–2021	7.5
Bele	6°55′40.9″N	37°31′33.0″E	1240	1987–2021	11.3
Bilate	6°49′00.1″N	38°04′59.9″E	1498	1987–2021	14
Bilate Tena	6°55′00.1″N	38°07′00.1″E	1482	1987–2021	8.5
Bodity	6°57′13.2″N	37°57′18.0″E	2233	1987–2021	12
Dana	6°38′07.1″N	37°34′15.6″E	1295	1987–2021	15
Halale	6°45′00.3″N	37°20′28.7″E	2254	1987–2021	18.5
Humbo Tebela	6°42′06.6″N	37°45′33.6″E	1628	1987–2021	18.75
Gesuba	6°43′26.4″N	37°33′27.6″E	1500	1987–2021	12
Wolaita Sodo	6°48′36.0″N	37°43′48.0″E	1854	1987–2021	10

Note: For all weather variables (rainfall), the record period is comparable for each station. The percentage of missing data denotes the percentage of missing data for each variable.

### 2.3 Data types and sources

The secondary data utilized in this study were sourced from the Ethiopian National Meteorological Agency, encompassing 34 years of monthly rainfall records from stations within the Wolaita zone region (1987–2021). The Ethiopian National Meteorological Agency (NMA) compiled daily rainfall data spanning from 1987 to 2021. The investigation utilized observed daily rainfall data obtained from NMA sites. Missing values for each rain gauge station analyzed in this study amounted to less than 20 % of the total missing daily and monthly rainfall data for a specific time series, as recommended by Campozano et al. [63]. However, rain gauge stations with more than 20 % missing rainfall data resulted in a considerable bias in the statistical analysis (Table 1).

To determine the % of missed data for each rain gauge station as the following method was used:

(1) $$\%\;missing\;data=\frac{\sum_{t=1}^{n}Missing\;data}{\;\sum_{t=1}^{n}\left(Observed\;available\;data+Missing\;data\right)}*100$$

where t is a given time interval (from $t_{1}tot_{n}$)

### 2.4 Methods of data analysis

#### 2.4.1 Filling missing data

The collected data were reviewed for quality, consistency, completeness, and errors or inconsistencies addressed. The missing data at stations had been substituted via a multiple imputation technique known as Markov Chain Monte Carlo (MCMC). Consequently, when it comes to filling in missing data, the MCMC algorithm outperforms alternative methods such as linear regression (MLR) and inverse distance weighting (IDW) [64,65]. The data were used for further analysis following the verification of these parameters. Imputation was performed using a Monte Carlo simulation of the MCMC method. The expectation-maximization (EM) technique [66] finds the maximum likelihood estimates for the MCMC method to replace the missing data. Multiple imputations fill in the missing values by creating plausible numbers based on the observed variable distributions and connections.

Van Buuren & Groothuis-Oudshoorn [67] and De Carvalho et al. [64] assert, let *Y* represent the complete dataset of a partially observed random sample, characterized by a multivariate distribution with p variables, denoted as P(*Y*|θ). The parameter vector θ, which is unknown, fully specifies the multivariate distribution of *Y*. The task is to ascertain the multivariate distribution of θ. The iterative process involves generating the posterior distribution of θ by sequentially sampling the conditional distributions of P(Y1|Y-1, θ1), …,P(Yp|Y-p, θp). The parameters θ1 … θp correspond to conditional densities and may not necessarily lead to the factorization of the actual joint distribution P(*Y*|θ).

When missing data are missing at random, meaning that the absence of certain values in a variable can be predicted from other variables in the dataset, a sample from the estimated marginal distribution is initially removed. The tth iteration of the linked equations involves sampling successively removed Gibbs samples.

(2) $$\begin{array}{cc} \theta_{p}^{*\left(t\right)}∼P(\theta_{P}\backslash Y_{1}^{obs},Y_{2}^{\left(t-1\right)}\text{,} & Y_{p}^{\left(t-1\right)}\\ Y_{1}^{*\left(t\right)}∼P(Y_{1}\backslash Y_{1}^{obs},Y_{2}^{\left(t-1\right)}\text{,} & Y_{p}^{\left(t-1\right)},\theta_{1}^{*\left(t\right)}\\ \begin{array}{c} \vdots \\ \begin{array}{c} \theta_{p}^{*\left(t\right)}∼P(\theta_{P}\backslash Y_{p}^{obs},Y_{1}^{\left(t\right)},\\ Y_{p}^{*\left(t\right)}∼P(Y_{p}\backslash Y_{p}^{obs},Y_{1}^{\left(t-1\right)}, \end{array} \end{array} & \begin{array}{c} Y_{p-1}^{\left(t\right)}\\ Y_{p}^{\left(t\right)},\theta_{p}^{*\left(t\right)} \end{array} \end{array}$$

where *Y*_j_^(t)^ = (Y_j_^obs^, *Y*_j_ *^(t)^), is the j^th^ parameter interpolated in iteration t. Due to the link with other variables, only *Y*_j_^(t−1)^ is taken into account when imputation of *Y*_j_ *^(t)^. θ*_p_ is represents the parameter estimate for the tth iteration of the chained equations.

#### 2.4.2 Weighted average rainfall estimation

**Fig. 3:**
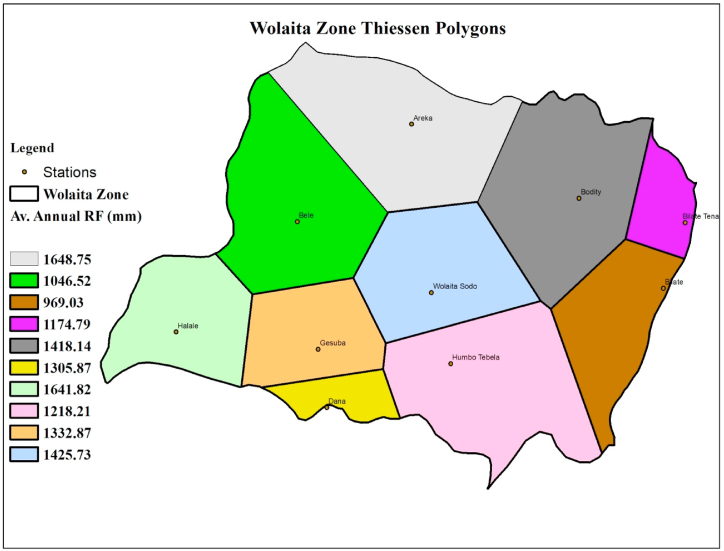
To estimate the weighted total rainfall for the region, Wolaita zone thiessen polygons are created from meteorological stations.

The areal weighted average annual and seasonal rainfall was computed using the Thiessen polygon method, and Lambe and Kundapura [71], utilized the same methodology for the complete area of the study basin provided by:

(3) $$Ȑ=\frac{\sum_{i=1}^{n}A_{i}R_{i}}{A}$$

where $A_{i}$ is the area of influence in the basin with a particular station (km^2^), A is the total area of the study area (km^2^), Ȑ is the weighted average of rainfall (mm), and $R_{i}$ is the average rainfall for stations (mm), and a method of assigning aerial significance to point rainfall values. Polygons were formed by cutting perpendicular bisectors along the lines that connected each point of measurement to the stations nearest to it. These bisectors interact to form a set of polygons, each with a single station. The names in the polygons show how much of the basin area the selected stations' names cover (Fig. 3).

#### 2.4.3 Rainfall trend analyses by temporal

I. Serial Correlation

Prior to trend analysis, the serial correlation test was performed on time series data (1987–2021) to ensure that observations were independent from year to year and prevent the autocorrelation effect [67]. At lag 1, the autocorrelation function (ACF package) [68,88] was utilized to determine whether the*XLSTAT with R programming* packages had any substantial autocorrelation. To calculate Lag 1 ACFs, the following formula was used:

(4) r1=(1n−1∑i=1n−1(xi−X®)(xi+1−X®))1n∑i−nn(xi−X®)2

In the context where the correlation coefficient at lag 1, the rainfall time series, and the mean value are denoted as r_1_, *X*_i_, and $\bar{X}$, respectively, the subsequent formula was utilized to derive the confidence interval for r_1_ at the 5 % significance level:

(5) $$r_{1}\left(5\%\right)=\frac{-1\pm 1.96\surd \left(n-1\right)}{n-1}$$

In cases where ‘n' represents the total number of observations in the time-series, the trend is examined using the Mann-Kendall (MK) trend test when ‘r_1_’ falls within the confidence interval and there is no significant autocorrelation. To account for autocorrelation effects, the Modified Mann-Kendall (MMK) test is applied, and if ‘r_1_’ falls outside the confidence interval, it indicates the presence of correlation among the corrected observations [69]. Due to the presence of both independence and serial correlation in the rainfall time-series analysis conducted with the *XLSTAT with R programming* software program, this study utilized both the Mann-Kendall (MK) and Modified Mann-Kendall (MMK) methods [70,91].

II. Temporal Trend Analyses

Seasonal and annual patterns in rainfall were investigated. Nonparametric trend analysis techniques such as Sen's slope estimates [72] and Mann-test Kendall's [73] are used to look for temporal patterns in rainfall.

#### 2.4.4 The Mann–Kendall's test

As per Croux and Dehon [74], this method is employed to examine the association between rankings and sequences within a time series. For a given time series, it computes the discrepancy between all preceding measurements and subsequent ones. The MK statistic 'S' increments by 1 when a later data point surpasses an earlier one, while it decrements by 1 if an earlier sampled data point exceeds a later one. The final value of '*S'* is determined by summing the values obtained from each scenario using the following formula:

(6) $$s=\sum_{i=1}^{N-1}\sum_{J=i+1}^{N-1}sgn\;\left(x_{j}-x_{i}\right)\text{,}$$

In this equation, 'S' denotes the Mann-Kendall test statistic, where x_i_ and x_j_ represent consecutive data values of a time series at years i and j (where j > i), respectively, and 'N' is the length of the time series. A positive '*S'* value indicates a rising trend, while a negative value indicates a declining trend in the data series. To distinguish between various trend types, the sign function was employed and is defined as:

(7) $$\text{sign}\left(x-j\right)\left(x-i\right)=\left\{ \begin{array}{c} +1,if\;\left(x_{j\;}-x_{i}\right)>0,\\ \;0,if\;\left(x_{j}-x_{i}\right)=0,\\ -1,if\left(x_{j}-x_{i}\right)<0 \end{array}\right.$$

For samples with n ≥ 10, the S statistic seems to be a normally distributed statistic, with a mean of zero and variance, as shown below [72];

(8) $$\sigma^{2}=\frac{1}{n}\left[n\left(n-1\right)\left(2n+5\right)-\sum_{i=1}^{m}\left(t_{1}-1\right)\left(2t_{i}+5\right)\right]$$

where, 'n' is the total number of observations in the time series, 'm' is the frequency of i^th^ groups, and t_i_ is the frequency of observations in the ith group.

'Z' score following compute the significance of a trend formula was used:

(9) $$Z=\left\{ \begin{array}{c} \frac{s-1}{\sigma },if\;s>0,\\ 0,if\;s>0,\\ \frac{s+1}{\sigma }if\;s>0 \end{array}\right.$$

At a selected level of significance α, the hypothesis of no trend is rejected if the 'Z' value exceeds the critical value 'Zα'.

#### 2.4.5 Sen's estimator of slope

The slope estimator (β) measures the magnitude of a linear trend and assesses temporal changes, indicating the existence of a trend. This method is robust against missing data and remains unaffected by outliers or significant errors [75]. Sen's approach [76] was employed to compute the slope (rate of change over time). Sen's slope (β) represents the median of slopes between pairs of points in a time series, with measurements taken at consistent intervals [77]. The calculation is as follows :

(10) $$\beta =\text{median}\frac{x_{i}-x_{j}}{i-j}\;i=1,2,3\ldots .\ldots \;n$$

In this context, X_i_ and X_j_ denote data values at ti and tj (where i > j) respectively. A positive β value indicates an “upward trend,” signifying increasing values over time, while a negative value denotes a “downward trend,” indicating decreasing values over time [37].

Tau [78] evaluates the importance of the monotonic relationship between x and y. Thus, the Kendall's tau correlation coefficient is expressed as:

(11) $$\tau =\frac{s}{n\left(n-1\right)/2}$$

A positive value of τ indicates an upward tendency and vice versa. The sum of the Mann-Kendall test statistic (*S*) reveals the strength of the rainfall trend and whether it is growing or decreasing.

## 3 Results and discussion

### 3.1 Spatial distribution of rainfall

Annually, there exists considerable geographic variability in rainfall distribution across AEZs. Throughout the study period, the average annual rainfall in the AEZs of the study area ranged from 969.03 mm in the southeast lowland AEZ to 1648.75 mm in the northwest highland AEZ. During the Kiremt season, precipitation levels varied from 772.53 mm in the northeast highland AEZ to 312.67 mm in the northwest lowland AEZ, constituting 29.87 %–54.48 % of the average annual rainfall. Conversely, the Bega season witnessed rainfall levels ranging from 326.04 mm in the northwest highland AEZ to 140.26 mm in the southeast lowland AEZ. Highland AEZs exhibited higher rainfall during both the short and main rainy seasons, while the south-central midland AEZ received an intermediate amount. In contrast, the northern regions of the east lowland and southeast lowland AEZs experienced diminished precipitation levels throughout the year compared to other areas.

**Fig. 4:**
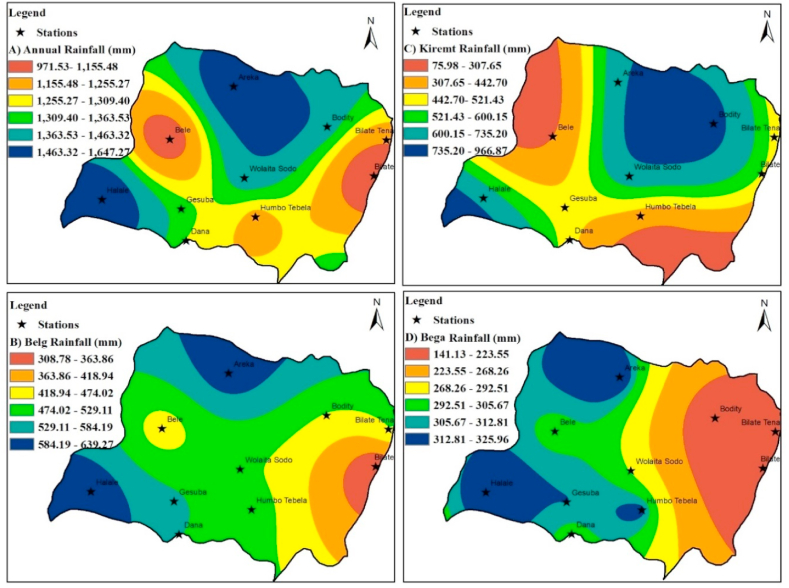
The spatial and seasonal/annual rainfall distribution over Wolaita zone (1987–2021).

The lowland AEZs, predominantly situated in the western, southern, and eastern sectors of the study domain, are characterized by warmer temperatures and reduced rainfall, while the highlands exhibit cooler climates and higher precipitation levels. This observation aligns with previous investigation by Wedajo et al. [79], who similarly noted the contrasting climatic conditions between lowland and highland areas. Miheretu [43], and Funk et al. [80] found that regions of higher elevation in Ethiopia tend to receive greater rainfall compared to low-arid areas, thereby supporting more substantial agricultural livelihoods and population densities. Moreover, rainfall variability, as evidenced by significant inter-annual fluctuations, exerts notable influences on crop productivity across various agricultural domains, a phenomenon corroborated by Kassie, [81] in the context of the rift valley's central region Fig. 4.

The results of ordinary kriging indicate that the mean annual rainfall coefficient of variability (CV) varies across different zones. In the southwest lowland AEZ, there was moderate variability, with a CV of 24.22 %, whereas in the northeast highland AEZ, there was higher variability, with a CV of 31.63 %. Moderate variability (25.35 %) was observed in the northeast lowland AEZ during the dry Bega season, while higher variability (60.91 %) was observed in the northeast highland AEZ. The southern lowlands experienced moderate variability (24.22), whereas the central midland AEZs showed higher variability. In the short rainy Belg season, moderate variability (25.77 %) was observed in the south-central midland AEZ, whereas the northeast highland AEZ had higher variability (46.25 %). This finding contrasts with Sahilu et al. [57] findings, who identified moderate rainfall variability (CV <30 %) in the highland areas, with values of 28 %, 30 %, and 29 % during the spring, summer, and autumn seasons, respectively. Notably, Birara [39] reported conflicting results, indicating that regions with low annual rainfall exhibited high rainfall coefficient of variability. It is essential to recognize that the association between high annual rainfall and rainfall variability may not universally apply and can vary depending on local factors such as climate and environment [82]. This finding aligns with Ware et al. [46] satellite imaging-based study in the Sidama region, which revealed that spatial and temporal variations in annual and seasonal rainfall are evaluated using coefficient variation (CV). Over a period of more than 30 years (1991–2020), the CV indicates that seasonal rainfall variability is significantly greater than annual rainfall variability. Consequently, previous research can serve as a basis for future investigations employing similar or comparative methodologies.

### 3.2 Trends of rainfall across stations in wolaita zone

The annual rainfall analysis showed negative trends at four stations (Areka, Bodity, Halale, and Humbo Tebela) and positive trends at the remaining six stations (Bele, Bilate, Bilate Tena, Dana, Gesuba, and Wolaita Sodo) (Table 2) Fig. 6. Belg rainfall exhibited negative trends at five stations (Areka, Bele, Bodity, Halale, and Humbo Tebela) and positive trends at the other five stations (Bilate, Bilate Tena, Dana, Gesuba, and Wolaita Sodo). Similarly, the Kiremt rainfall showed negative trends at six stations (Areka, Bilate, Bodily, Dana, Halale, and Gesuba) and positive trends at the remaining three stations (Bele, Bilate Tena, and Wolaita Sodo), while at one station, there was no change (Humbo Tebela) (Fig. 7).

**Table 2:** Station-based rainfall trends in Wolaita zone (1987–2021).

Station	AEZs	Annual			Belg			Kiremt			Bega		
		τ	*p*-value	β	τ	*p*-value	β	τ	*p*-value	Β	τ	*p*-value	β
Areka	Midland	−0.207	0.083	−16.260	−0.167	0.160	−5.273	−0.316	0.008***	−9.859	0.052	0.673	0.546
Bele	Lowland	−0.005	0.966	0.000	−0.148	0.211	−3.077	0.059	0.619	1.465	0.025	0.831	0.405
Bilate	Lowland	0.020	0.865	1.506	0.029	0.809	0.879	−0.189	0.112	−5.273	0.324	0.006***	5.275
Bilate Tena	Lowland	0.264	0.026***	16.664	0.148	0.211	4.963	0.034	0.776	0.811	0.435	0.000***	8.336
Bodity	Highland	−0.244	0.039***	−16.653	−0.179	0.132	−4.519	−0.333	0.005***	−10.750	0.063	0.599	1.014
Dana	Lowland	0.096	0.418	4.284	0.109	0.356	2.102	−0.130	0.274	−3.245	0.356	0.003***	5.524
Halale	Highland	−0.200	0.094	−16.770	−0.170	0.151	−5.260	−0.316	0.008***	−9.903	0.047	0.691	0.352
Humbo Tebela	Midland	−0.022	0.853	−1.054	−0.105	0.378	−1.846	0.002	0.989	0.000	0.165	0.164	2.344
Gesuba	Lowland	0.034	0.776	1.406	0.039	0.744	0.754	−0.131	0.268	−3.650	0.268	0.024***	3.836
Wolaita Sodo	Midland	0.200	0.094	10.959	0.005	0.978	0.100	0.210	0.078	4.889	0.287	0.015***	6.940

Significant trends at α = 0.05 are shown by bold values, Sen's slope, and Kendall's tau are, β and τ respectively.

**Fig. 5:**
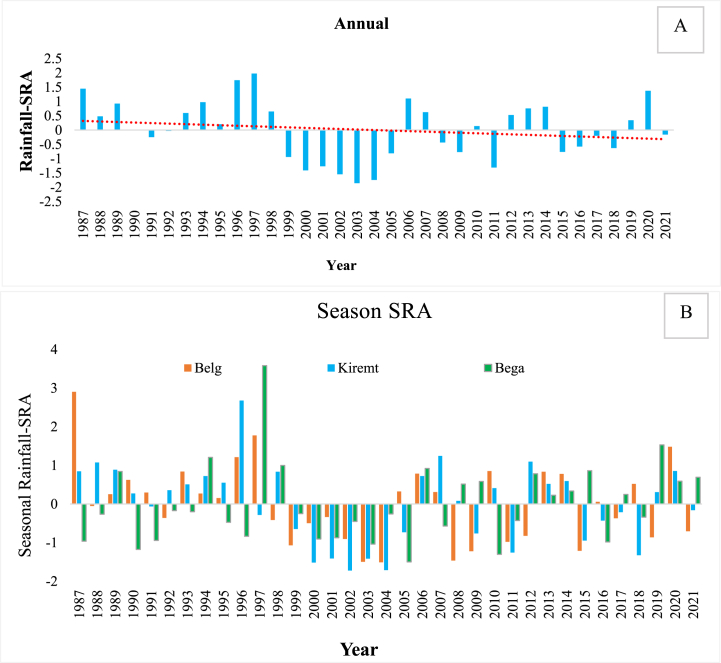
Trends of annual rainfall across stations.

Severe wet conditions were seen in 2019 and 1997 (2.86 %) during the Bega and Belg seasons, respectively; there were no severe wet years during the Kiremt season. Moderately wet conditions were seen in 1994 (2.86 %) of the examined years during Bega and 1988, 2007, and 2012 (8.57 %) during the analyzed years during the Kiremt season, as well as in 1996 and 2020 (5.71 %) during the Belg season (Fig. 5 A and B).

On the weighted rainfall in area other side, the negative anomaly revealed that the Wolaita zone had moderately and severely dry years. Severe dry conditions were reported throughout the Belg and Kiremt seasons, accounting for 2.86 % (2004) and 8.57 % (2000, 2002, and 2004), respectively. The findings revealed that 11.43 % of the examined years (1990, 2003, 2005, and 2010) had moderate dryness in the Bega season, similar to Kiremt, which accounted for 11.43 % of total years (2001, 2003, 2011, and 2018).

**Fig. 6:**
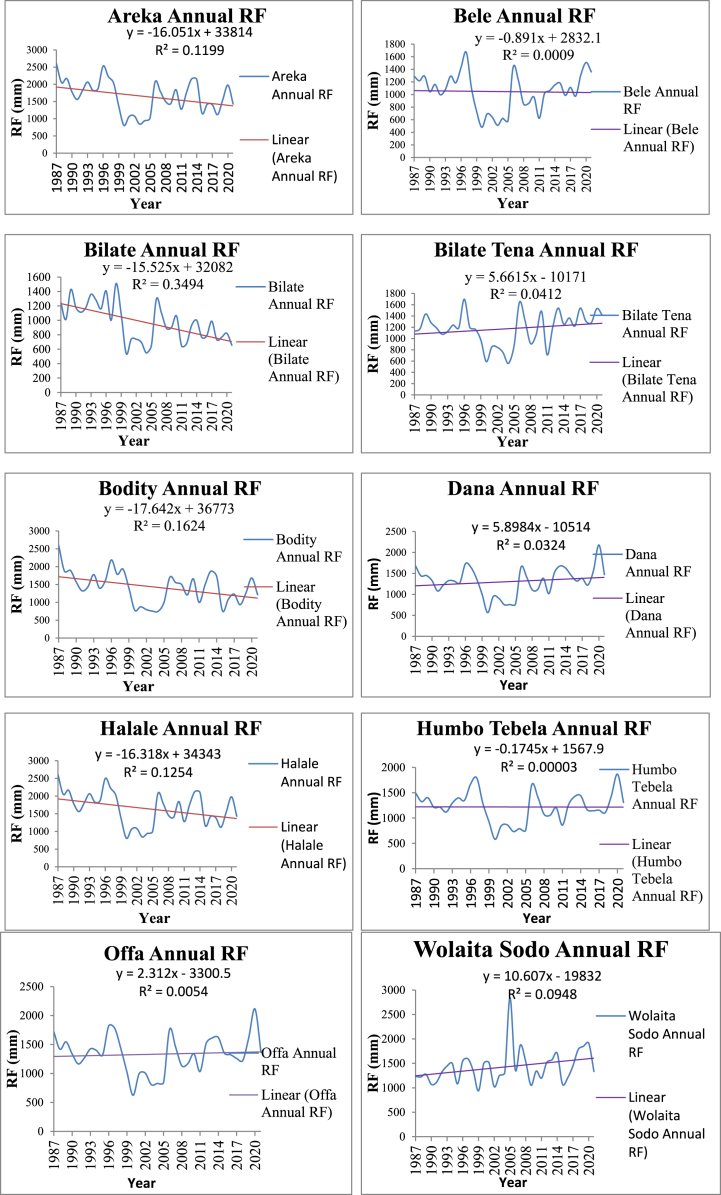
The MK test revealed the spatial variance of rainfall trends at the 5 % significance level for the a) Annual, b) Belg, c) Kiremt, and d) Bega periods.

**Fig. 7:**
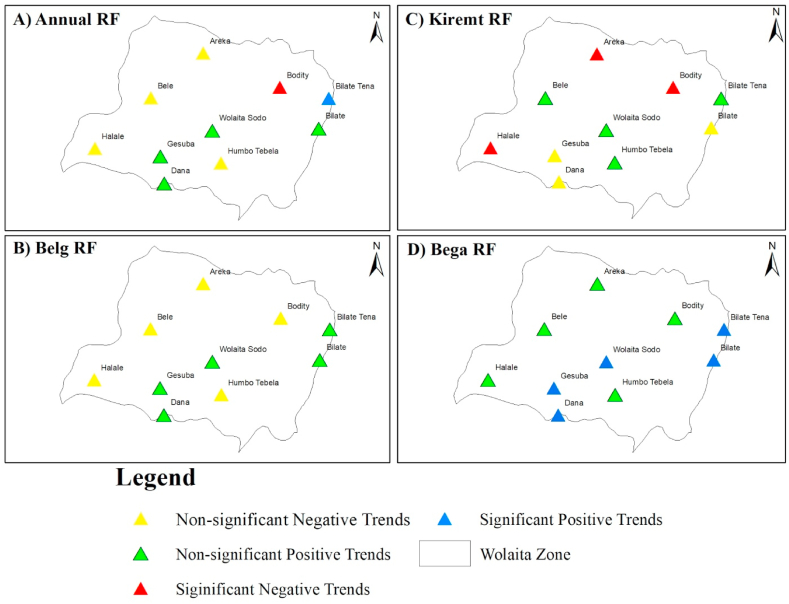
The spatial variation of a) Annual; b) Belg; c) Kiremt and d) Bega timescales of rainfall trends identified by the MK test at 5 % significance level.

This study's findings contradict Alemu and Bawoke [83], who discovered that the percentage of negative anomalies exceeded that of positive anomalies in all seasons except Kiremt in the Amhara area. This might be due to variances in the study area's spatial position and the approaching rain front. This study, which is consistent with Zegeye et al. [24], demonstrated that roughly 11.4 % of the examined years (1984, 1990, 1995, and 2005) were moderately dry in the Bega season, and 5.71 % in 1997 and 2015. The Bega season saw a higher number of negative anomalies (57.14 %). This suggests that in the worst-case situation, stream flows and groundwater levels might plummet. Negative anomalies indicate a potential decline in stream flows and groundwater levels, posing a significant danger to irrigated agriculture and related industries [93]. According to Sahilu et al. [57], short-term fluctuations are caused by changes in El Niño (ENSO), Indian Ocean dipole (IOD), and differential warming over the Indian Ocean.

The annual rainfall ranges from 1.406 mm/year at Gesuba to 16.664 mm/year at Bilate Tena, indicating an increasing or positive Sen's slope estimate; the decreasing/negative magnitude is from −16.770 mm/year at Halale to −1.054 mm/year at Humbo Tebela stations. Table 2 revealed that only 50 % of the stations showed substantial increases in the Bega season, whereas none of the stations showed significant increases in the Kiremt or Belg seasons. Only one station (Bilate Tena) at the study station had a statistically significant (*p* < 0.05) positive change in the annual rainfall, and only one station (Bodity) demonstrated a statistically significant (*p* < 0.05) negative change. Three stations (Areka, Bodity, and Halale) in the Kiremt session demonstrated a considerable decline, whereas the other stations showed no discernible change. Half of the sites had a statistically significant increase (*p* < 0.05) during the Bega session (Table 2).

In accordance with Lambe and Kundapura [71], this study observed no significant changes in rainfall patterns during the Belg season across all stations. Previous research has identified modest (p > 0.05) and notably declining (α = 0.1) trends during the Kiremt season at the Alaba Kulito and Bilate Tena stations, respectively [31]. In Rift Valley basins, there is a 2.9 mm/year decrease in annual rainfall and a minor increasing trend of 0.9 mm/year during the Belg season [35]. The majority of stations exhibited minimal trends, as per Addisu et al. [49], with a combination of insignificant and significant downward annual trends in the Tana Basin [39], and non-significant trends in the upper wash basin.

According to Ratner [89], the correlation coefficient is assessed on a scale from +1 to −1, with a complete correlation represented by +1 or −1. A positive correlation occur when one variable increases and a negative correlation when one variable decreases. The annual rainfall *R*^2^ in the study area ranges from a minimum of 0.00003 at Humbo to 0.3494 at the Bilate Station. These findings suggest a slight positive correlation between the year and annual rainfall across stations in the Wolaita zone. This implies that annual rainfall variations, as independent variables, contribute between 0.003 % and 34.94 % to the dependent variable, year in time. The linear regression line in Fig. 5 indicates an overall upward trend in annual rainfall for the three study districts, albeit with varying magnitudes. Similarly, the study observed a positive slope for the two agriculturally significant rainy seasons (Kiremt and Belg) across all study districts.

### 3.3 Spatial mapping of annual and seasonal rainfall trends by MK

The MK was used to determine the spatial distributions of the seasonal and annual rainfall trends. Understanding the effects of regional and global climate change as well as climatic variability on current and future water supplies has been accomplished through the use of spatial mapping of trend analysis results [31]. Rainfall trend analysis for the 1987–2021 periods was conducted to identify recent changes in rainfall patterns in the Wolaita zone AEZs. Analysis of annual rainfall in different agro-ecologies revealed that most lowland AEZs exhibited an upward or positive trend.

The findings showed that statistically significant positive trends were observed in a significant percentage (20 %) of the lowland stations in Wolaita. Moreover, 20 % of the stations in the same AEZ displayed declining trends that were not statistically significant (*p* > 0.05). On the other hand, non-significant positive trends for yearly rainfall were seen at 60 % of the lowland AEZ stations. Fig. 6 indicates that 50 % of highland stations exhibited statistically non-significant (*p* > 0.05) negative trends for annual rainfall, whereas 50 % showed statistically significant (*p* < 0.05) negative trends.

In the highland AEZ, 66 % of the stations experienced non-significant negative trends and the remaining 33.33 % showed non-significant (*p* > 0.05) positive trends. It is noteworthy that the highland stations (Bodity station) demonstrated a statistically significant (*p* < 0.05) decline in the total annual rainfall over the lengthy period of time for each station. In contrast, Fig. 7 indicates that the total annual rainfall at the zonal level increased in a statistically significant (*p* < 0.05) manner in the lowland sections of Bilate Tena.

During the Belg season, all highland AEZs had statistically non-significant (*p* > 0.05) negative trends, two-thirds of midland AEZs had statistically non-significant (*p* > 0.05) negative trends, and four-fifths of lowland AEZs had a statistically non-significant positive trend. During the Kiremt session for each AEZ, all highland regions (Bodity and Halale) and 1/3 of the midland stations had a substantial decline (*p* < 0.05) in total annual rainfall. The remaining AEZs exhibited statistically insignificant positive and negative trends (*p* > 0.05). During the Bega season, 80 % of the lowlands and 33.33 % of the midlands experienced a significant positive trend. The other AEZs experienced statistically non-significant (*p* > 0.05) positive patterns during Kermit sessions (Fig. 7).

The findings of our study align with those reported by Gummadi et al. [32], emphasizing significant changes in rainfall volume and distribution within the study area. These observed patterns exhibit considerable spatiotemporal variability across an extensive spatial domain, underscoring their robustness. Importantly, these trends may serve as a foundational step toward comprehending broader climate dynamics and water resource variations [32]. This inference contradicts Weldegerima et al. [87], who discovered that a study of rainfall patterns indicates that the amount of annual rainfall in the Lake Tana basin is increasing, albeit at a non-statistically significant rate. Seasonal studies indicate that the Bega season receives the least amount of rainfall, and that this season is growing drier with time.

Building upon this context, additional insights emerge from related research. Harka et al. [84], Sharma and Singh [85], and Alemu and Bawoke [83], emphasize the critical role of spatial variability in rainfall patterns. Specifically, this variability holds implications for impact assessment and adaptation planning, particularly concerning floods, droughts, and extreme events.

Our study contributes valuable trend data for three distinct seasons, delineated by AEZs. These data provide a lens through which to evaluate the potential consequences of shifting rainfall patterns. Moreover, they inform the development of context-specific adaptation strategies, tailored to the unique challenges posed by specific seasons and local contexts [86,90].

## 4 Conclusion and policy implication

A GIS-based spatiotemporal investigation of fluctuations in rainfall under climate change in several AEZ zones in the Wolaita zone, South Ethiopia, provides useful insights into the region's changing precipitation patterns. This study not only emphasizes the need to use GIS technology to monitor and analyze climatic factors, but also provides useful information for policymakers and agricultural practitioners seeking to build potential adaptation measures. The fundamental contribution of this study is its thorough examination of rainfall patterns across numerous AEZs, which sheds light on the various effects of climate change on agricultural practices in Wolaita. By combining spatial and temporal finding, this study improves our understanding of the changing climatic dynamics of the region, ultimately driving sustainable resource management and resilience-building initiatives. This study conducted a spatiotemporal analysis of rainfall trends under climate change in various AEZs in Wolaita, South Ethiopia. MK and Sen's slope analyses were used to estimate annual and seasonal rainfall trends. For spatial interpolation, Kirging emphasizes geo-statistical interpolation. The current study indicated that it is necessary to understand the influencing mechanism systematically and comprehensively based on its findings as well as on detailed literature. More rainfall was received by the highland AEZs in spatial allocation of mean annual rainfall and seasonal (short and main rainy seasons) rainfall (<2000 mm) than the south-central midland (1200–1600 mm). The annual temporal trends were assessed over the three AEZs. Majority of lowland AEZsannual rainfall trended upward direction. The finding revealed considerable variability in the rainfall patterns across the AEZs over the study period. The AEZ, categorized as highland, exhibited the most significant decreasing trend in annual rainfall, followed by the midland and lowland zones. These findings suggest that climate change has started impacting rainfall patterns in the Wolaita zone, with potentially adverse effects on agricultural production and water resources.

The results also emphasize the importance of incorporating GIS-based spatiotemporal analysis in climate change investigations to better understand the complex interactions between climate and agriculture. These findings revealed a distinct pattern of rainfall changes across various AEZs at the Wolaita zone level, highlighting the importance of implementing tailored adaptation strategies to mitigate the effects of significantly changing precipitation patterns on agricultural productivity.

To address these issues, we recommend:

• Concerned entities should priorities developing sustainable water management methods to improve farmer resilience to shifting rainfall patterns.
• To reduce the impact of unpredictable precipitation on agricultural productivity, governments and non-governmental organizations should encourage crop diversity and soil-water conservation strategies for specific AEZs.
• To support farmers and policymakers in making timely decisions, it is imperative to strengthen weather monitoring and early warning systems in the various AEZ of the Wolaita zone, south Ethiopia. It is crucial to work towards improving the ability of pertinent stakeholders to use this information for sustainable agriculture practices and decision-making.

Based on this finding, future researchers should focus on conducting more detailed studies to investigate the underlying factors driving rainfall trends in various AEZs , as well as investigating the potential of using machine learning and remote sensing technology to make more accurate and timely rainfall predictions. Furthermore, more investigation is needed to evaluate the potential and simulated efficiency of various adaptation measures for improving climate resilience and sustainable agriculture in the study region. For future research, further investigation into the specific factors driving these rainfall trends in Wolaita, as well as the development of more localized adaptation strategies, are recommended to build resilience in the face of ongoing climate change.

Finally, this study limited in the scope of “*Spatiotemporal Trends of Rainfall in Wolaita Zone*” in a given period. Understanding the microclimate of the study region demands additional investigation on the temporal and spatial patterns of temperature, as well as an evaluation of the rate of evaporation in each of the three AEZ in linking with regional and global climate model impact should be address. The conclusion will minimize the difficulties with smallholders' means of sustenance.

## Availability of data and materials

The datasets generated and analyzed during the current study are included in the body of this paper.

## Funding

The study received no funding from government, commercial, or non-profit financing organizations.

## CRediT authorship contribution statement

**Elias Bojago:** Writing – review & editing, Writing – original draft, Visualization, Validation, Software, Resources, Project administration, Methodology, Investigation, Funding acquisition, Formal analysis, Data curation, Conceptualization. **Ayele Tessema:** Supervision, Writing – review & editing. **Innocent Ngare:** Writing – review & editing, Validation, Resources.

## Declaration of competing interest

The authors declare that they have no known competing financial interests or personal relationships that could have appeared to influence the work reported in this paper.
